# Actin-Mediated Gene Expression Depends on RhoA and Rac1 Signaling in Proximal Tubular Epithelial Cells

**DOI:** 10.1371/journal.pone.0121589

**Published:** 2015-03-27

**Authors:** Klaudia Giehl, Christof Keller, Susanne Muehlich, Margarete Goppelt-Struebe

**Affiliations:** 1 Signal Transduction of Cellular Motility, Internal Medicine V, Justus-Liebig-University Giessen, Giessen, Germany; 2 Department of Nephrology and Hypertension, Friedrich-Alexander Universität Erlangen-Nürnberg, Erlangen, Germany; 3 Walther Straub Institute of Pharmacology and Toxicology, Ludwig-Maximilians-University, Munich, Germany; Beatson Institute for Cancer Research Glasgow, UNITED KINGDOM

## Abstract

Morphological alterations of cells can lead to modulation of gene expression. An essential link is the MKL1-dependent activation of serum response factor (SRF), which translates changes in the ratio of G- and F-actin into mRNA transcription. SRF activation is only partially characterized in non-transformed epithelial cells. Therefore, the impact of GTPases of the Rho family and changes in F-actin structures were analyzed in renal proximal tubular epithelial cells. Activation of SRF signaling was compared to the regulation of a known MKL1/SRF target gene, connective tissue growth factor (CTGF). In the human proximal tubular cell line HKC-8 overexpression of two actin mutants either favoring or preventing the formation of F-actin fibers regulated SRF-mediated transcription as well as CTGF expression. Only overexpression of constitutively active RhoA activated SRF-dependent gene expression whereas no effect was detected upon overexpression of Rac1 mutants. To elucidate the functional role of Rho kinases as downstream mediators of RhoA, pharmacological inhibition and genetic inhibition by transient siRNA knock down were compared. Upon stimulation with lysophosphatidic acid (LPA) Rho kinase inhibitors partially suppressed SRF-mediated transcription, whereas interference with Rho kinase expression by siRNA reduced activation of SRF, but barely affected CTGF expression. Together with the partial inhibition of CTGF expression by the pharmacological inhibitors Y27432 and H1154, Rho kinases seem to be less important in mediating RhoA signaling related to CTGF expression in HKC-8 epithelial cells. Short term pharmacological inhibition of Rac1 activity by EHT1864 reduced SRF-dependent CTGF expression in HKC-8 cells, but was overcome by a stimulatory effect after prolonged incubation after 4-6 h. Similarly, human primary cells of proximal but not of distal tubular origin showed inhibitory as well as stimulatory effects of Rac1 inhibition. Thus, RhoA signaling activates MKL1-SRF-mediated CTGF expression in proximal tubular cells, whereas Rac1 signaling is more complex with adaptive cellular responses.

## Introduction

The small GTPases RhoA and Rac1 are major regulators of cell morphology by modulating fibrous actin (F-actin) structures. The dynamic equilibrium between F-actin and monomeric actin triggers interactions of monomeric actin with various actin-binding proteins, among them the coactivator MKL1 (myocardin-related transcription factor 1, also known as MAL or MRTF-A), a binding partner of serum response factor (SFR) [1]. RhoA-induced actin polymerization has been shown to reduce monomeric actin which allows MKL1 to interact with serum response factor (SRF) and leads to upregulation of a subset of SRF-responsive genes [2]. The binding site of the MKL1-SRF complex, the CArG box element, closely resembles the SRE element, which mediates growth factor dependent activation of SRF, but does not contain the flanking Ets binding sites [3]. A CArG box-like element is also enclosed in the promoter of connective tissue growth factor (CTGF, CCN2) [4]. Expression of this matricellular protein has been proven to be particularly sensitive to all types of changes in actin cytoskeletal organization [5, 6]. Examples are upregulation of CTGF in endothelial cells upon shear stress [7] or in cardiomyocytes upon stretching [8].

Activation of RhoA—Rho kinases leading to SRF-mediated activation of CTGF synthesis has been shown by us and by others in various types of mesenchymal cells [6]. Far less is known about a link between Rac1, SRF and CTGF. Busche et al. provided evidence that in MDCK cells, renal tubular cells of distal tubular origin, activation of Rac1, but not RhoA is essential for SRF activation upon disruption of cell-cell adhesions [9]. However, CTGF as SRF target gene was not analyzed in those studies. Elevated Rac1 activity was reported in scleroderma fibroblasts, which are characterized by strong F-actin fibers [10]. In these cells, Rac1 was shown to be essential for the maintenance of the persistent fibrotic phenotype of the cells, including enhanced expression of CTGF. Thus far, the impact of both RhoA and Rac1 signaling has not been compared in one cell type in terms of CTGF induction.

The proximal tubules of the kidney consist of unique epithelial cells which instead of E-cadherin express N-cadherin as the most prominent cell-cell adhesion molecule [11]. When isolated from human kidneys these cells proved to be morphologically distinct compared to distal tubular cells, which express E-cadherin as major cell-cell adhesion molecule as do all other adult human epithelial cells [12]. Compared to E-cadherin expressing cells, proximal epithelial cells were less adherent, formed three-dimensional structures upon prolonged culture and were sensitive to TGF-β treatment. Pharmacological inhibition of Rho kinases, which are essential mediators of Rho-mediated alteration of F-actin fibers, reduced the expression of N-Cadherin, but not E-cadherin [12]. Inhibition of the Rho kinase isoforms, ROCK1 and ROCK2, differentially affected F-actin structures, most obviously in immortalized proximal cells (HKC-8 cells). These data suggested that Rho kinases might differentially affect actin-mediated modulation of SRF activity and target gene expression.

In the present study actin-dependent activation of SRF was compared to the activation of CTGF synthesis, which contains a MKL1-SRF dependent binding site in its promoter. Proximal renal tubular cells were chosen as model system because they show a higher morphological plasticity than other types of epithelial cells. The impact of changes in F-actin and the role of Rac1 and RhoA signaling as well as a potential role of individual ROCK isoforms was analyzed in immortalized and primary proximal tubular cells.

## Materials and Methods

### Materials

DMEM/Ham’s F12 medium was purchased from Biochrom AG (Berlin, Germany), DMEM medium and Hank´s BSS from PAA Laboratories (Coelbe, Germany), insulin-transferrin-selenium supplement from Gibco (Karlsruhe, Germany), fetal calf serum (FCS) from PAN Biotech (Aidenbach, Germany), triiodothyronine from Fluka (Buchs, Switzerland), hydrocortisone and lysophosphatidic acid (LPA) from Sigma-Aldrich (Munich, Germany), epidermal growth factor from PeproTech (Hamburg, Germany), Y27632, (+)-(*R*)-*trans*-4-(1-aminoethyl)-*N*-(4-pyridyl) cyclohexanecarboxamide dihydrochloride from Calbiochem, H1152 (*S*)-(+)-2-methyl-1-[(4-methyl-5-isoquinolinyl)sulfonyl]-homopiperazine from Alexis Biochemicals (Grünberg, Germany), and EHT1864 (5-(5-(7-(Trifluoromethyl)quinolin-4-ylthio)pentyloxy)-2-(morpholinomethyl)-4H-pyran-4-one dihydrochloride) from Sigma-Aldrich.

### Cell culture

HKC-8 cells were kindly provided by L. Racusen (Baltimore, MD) [13]. Cells were recloned by limited dilution and cultured as described previously [14]. Human primary tubular epithelial cells were isolated from renal cortical tissues collected from healthy parts of tumor nephrectomies essentially as described previously [11]. Isolation of human cells from healthy parts of tumor nephrectomies was approved by the local ethics committee (Reference number 3755, Ethik-Kommission der Medizinischen Fakultät der Friedrich-Alexander Universität Erlangen-Nürnberg). We obtained written informed consent from all participants involved in this study.

### Western blot analysis

Cells were lyzed in buffer containing 50 mM HEPES pH 7.4, 150 mM NaCl, 1% Triton X-100, 1 mM EDTA, 10% glycerol, 2 mM sodium vanadate and protease inhibitors complete EDTA-free (Roche Diagnostics, Mannheim, Germany) or in phosphate-buffered saline containing 5% SDS plus inhibitors to detect phosphorylated proteins. Equal volumes of cell culture supernatants were precipitated with ethanol to detect secreted CTGF. Western blot analyses were performed essentially as described before [14] using the following antibodies: mouse anti-vinculin (SC-5573), goat anti-CTGF (SC-14939), mouse anti-RhoA (SC-418), goat anti-MKL1 (SC-21558) and peroxidase-conjugated donkey anti-goat IgG (SC-2020), from Santa Cruz; mouse anti-Rac1 (#610651) from BD Transduction Laboratories; rabbit monoclonal anti-MYPT (YE336) from Epitomics; rabbit anti-phospho-ERK (#9106), mouse anti-ERK (#9107), rabbit anti-phospho-Cofilin (Ser3) (#3311) and rabbit anti-phospho-MYPT (Thr853) (#4563) from Cell Signaling, mouse anti-tubulin (T0198) from Sigma-Aldrich, sheep anti-mouse IgG (NA931V) and donkey anti-rabbit IgG (NA934V) from Amersham Biosciences.

To ensure equal loading and blotting, blots were redetected with an antibody directed against tubulin or vinculin. The immunoreactive bands were quantified using the luminescent image analyzer (LAS-1000 Image Analyzer, Fujifilm, Berlin, Germany) and AIDA 4.15 image analyzer software (Raytest, Berlin, Germany). To summarize data obtained from different cell cultures, relative band intensities were normalized as indicated in the legends.

### Immunocytochemistry

Cells were fixed with paraformaldehyde (3.5% in PBS) for 10 min and afterwards permeabilized with 0.5% Triton X-100 in PBS for 10 min. After washing three times with PBS, cells were blocked in 1% BSA in PBS for 1 h at room temperature and washed once.

Primary antibodies were those used for Western blotting. Secondary antibodies (1:500, PromoFluor 488 anti-mouse A21202 or 488 anti-rat A11006) were from Promokine. F-actin was stained with PromoFluor 488 or 555 phalloidin from PromoKine, nuclei were visualized with Hoechst (Sigma-Aldrich).

After mounting, slides were viewed using a Nikon Eclipse 80i fluorescent microscope and digital images recorded by Visitron Systems 7.4 Slider camera (Diagnostic Instruments, Puchheim, Germany) using Spot Advanced software (Diagnostic Instruments) or with the Keyence BZ-9000 system.

### DNA transfection

Cells were seeded on collagen IV-coated cover slips at low density (12,500 cells/cm^2^). The next day, cDNA constructs encoding constitutively active Rac1 (human pEGFP/Rac1(G12V)), dominant negative Rac1 (human pEGFP/Rac1(T17N), constitutively active RhoA (human pEGFP/RhoA(G14V)) or dominant negative RhoA ((human pEGFP/RhoA(T19N)) [15] were transfected using X-treme HD (Roche) or K2 Multiplier and K2 transfection reagent (Biontex Laboratories) following the manufacturers’ instructions.

A 4.5 kb CTGF promoter cloned into pGL3 was kindly provided by D. Abraham, University College, London, UK. pEF-actin S14C, pEF-actin R62D and p3D.A-Luc comprising three SRE with a mutated Ets motif were kindly provided by G. Posern, Martin Luther University Halle-Wittenberg, Germany [16–18].

### Determination of Rac1 activity

Rac1 activity was determined essentially as described previously [19]. The GTP-bound form of Rac1 was recovered from 500 μg of cell lysate by affinity precipitation using a GST-fusion protein carrying the Rac1 binding domain of PAK1B as an activation-specific probe for endogenous Rac1 [15].

### siRNA Transfection

siRNA transfections were performed essentially as described previously [12] To down-regulate ROCK1, ROCK2, MKL1 or RhoA epithelial cells were transfected 3 h after seeding using HiPerFect (QIAGEN GmbH, Hilden, Germany) according to the manufacturer’s instructions. siRNA directed against GFP was used as control. Experiments were performed 48 h after transfection. Silencing of Rho kinases by transient siRNA transfection was over 80% as determined by Western blotting [12]. An example is shown in S1A Fig. Silencing of RhoA (si: 5’ GAC AUG CUU GCU CAU AGU C) was 73 ± 5% (means ± SD of 3 experiments with duplicate transfections). An example is shown in S1B Fig. Silencing of MKL1 has been described in [20], (si 5’ GAA UGU GCU ACA GUU GAA A). In HKC-8 cells, down-regulation was over 95%. An example is shown in S1C Fig.

### Migrations assays

Migration assays were performed as described previously [21].

### Statistical analysis

To compare multiple conditions, statistical significance was calculated by one-way ANOVA with Dunnett’s or Tukey’s post hoc test, one sample or Student’s t-test using GraphPad software. A value of p< 0.05 was considered to indicate significance.

## Results

### Repression of gene expression by monomeric actin

**Fig 1:**
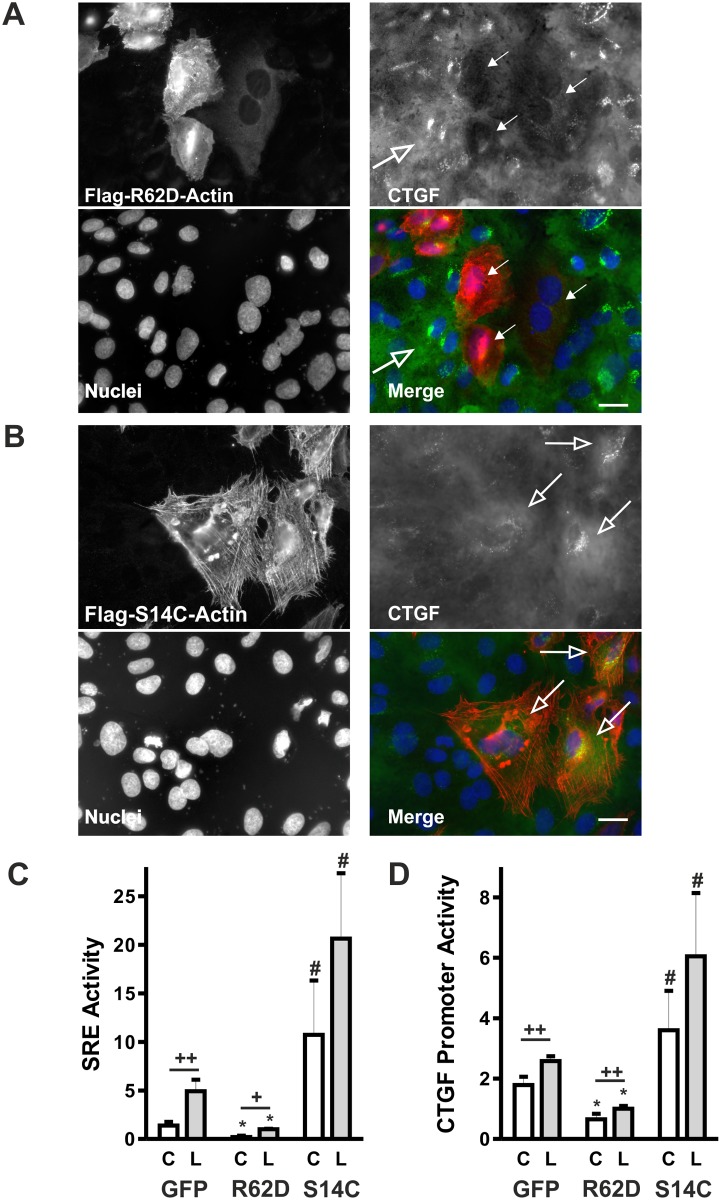
Actin mutants alter CTGF expression. (A) HKC-8 cells were transfected with the flag-tagged polymerization-defective actin mutant R62D. 24 h after transfection, cells were stimulated with LPA (10 μM) for 2 h. Mutated actin (anti-flag, red) and CTGF (green) were visualized by indirect immunofluorescence; nuclei were stained with Hoechst (blue). Open arrow indicates CTGF expression in a LPA-stimulated cell; closed arrows indicate low CTGF expression in actin R62D transfected cells. Scale bar: 20 μm. (B) HKC-8 cells were transfected with the flag-tagged polymerization favoring actin mutant S14C. Cells were fixed after transfection without further stimulation. Mutated actin (anti-flag, red) and CTGF (green) were visualized by indirect immunofluorescence; nuclei were stained with Hoechst (blue). Open arrows indicate CTGF expression in transfected cells. Scale bar: 20 μm. (C): HKC-8 cells were transfected with actin expression plasmids (actin R62D and S14C) or eGFP as control and with a luciferase-coupled promoter construct comprising three SRE elements. Expression of cotransfected beta galactosidase was used as reference. Cells were stimulated with LPA (L, 10 μM) for 4 h and compared to control cells (C). Data are means ± SD of 2–4 experiments with biological duplicates. SRE activity of LPA-stimulated actin R62D-transfected cells was set to 1. # p < 0.01 compared to the respective R62D actin- or eGFP-transfected cells; * p < 0.001 analyzed separately compared to eGFP-treated cells. ANOVA with Tukey’s multiple comparison test; ++ p<0.001, + p<0.05, analysis of eGFP and R62D treated cells, LPA-stimulated cells compared to control cells. (D) Cells were treated as in (C), but co-transfected with a 4.5 kb CTGF promoter construct. # p < 0.01 compared to the respective R62D actin- or eGFP-transfected cells; * p < 0.01 analyzed separately compared to eGFP-treated cells. ANOVA with Tukey’s multiple comparison test; ++ p<0.001, analysis of eGFP and R62D treated cells, LPA-stimulated cells compared to control cells.

Overexpression of mutated actin in the human proximal tubular cell line HKC-8 markedly altered cell morphology and gene expression. The polymerization-defective actin mutant R62D was localized primarily in the cytosol and induced rounding of the cells (Fig. 1A and S2 Fig.). By contrast, the actin S14C polymerization favoring mutant was incorporated into F-actin fibers and induced spreading of HKC-8 cells (Fig. 1B and S2 Fig.). As potential actin-dependent target gene, expression of CTGF was analyzed. To induce CTGF synthesis, cells were stimulated with lysophosphatidic acid (LPA), which is a known activator of RhoA-Rho kinase signaling [22]. Newly synthesized CTGF was detected in a perinuclear localization, whereas secreted CTGF was diffusely distributed over the cells (open arrow head in Fig. 1A). Expression of CTGF was suppressed in cells transfected by the polymerization-defective actin mutant R62D (Fig. 1A, closed arrows). Cells overexpressing the actin S14C polymerization-favoring mutant strongly expressed CTGF even in the absence of LPA (arrows in Fig. 1B).

Alterations of G-actin levels lead to increased binding or liberation of MKL1, an activator of the transcription factor SRF [2]. To address MKL1-mediated activation of SRF, cells were transfected with a construct comprising three serum response elements (SRE) with a mutated Ets motif. This element thus contains the CArG box and is selectively activated by MKL1-activated SRF and not by Ras-ERK-mediated activation of SRF [3]. Compared to eGFP-transfected cells, SRE activity was markedly increased in cells transfected with actin S14C and decreased in cells transfected with actin R62D (Fig. 1C, control cells, white bars).

Comparable to the activation of the SRE element, the activity of a construct comprising 4.5 kb of the human CTGF promoter (Fig. 1D), which contains a MKL1-SRF sensitive CArG box [4], reflected the alterations in G-actin levels. When G-actin levels were elevated by overexpression of actin R62D actin, promoter activity was reduced but was increased, when G-actin levels were reduced by forced F-actin polymerization in S14C actin overexpressing cells (Fig. 1D). Stimulation of the cells with LPA (L—grey bars) further increased SRE and CTGF promoter activity.

These data provided evidence for actin-dependent regulation of CTGF in proximal tubular epithelial cells.

### Activation of SRF-dependent gene expression by RhoA

**Fig 2:**
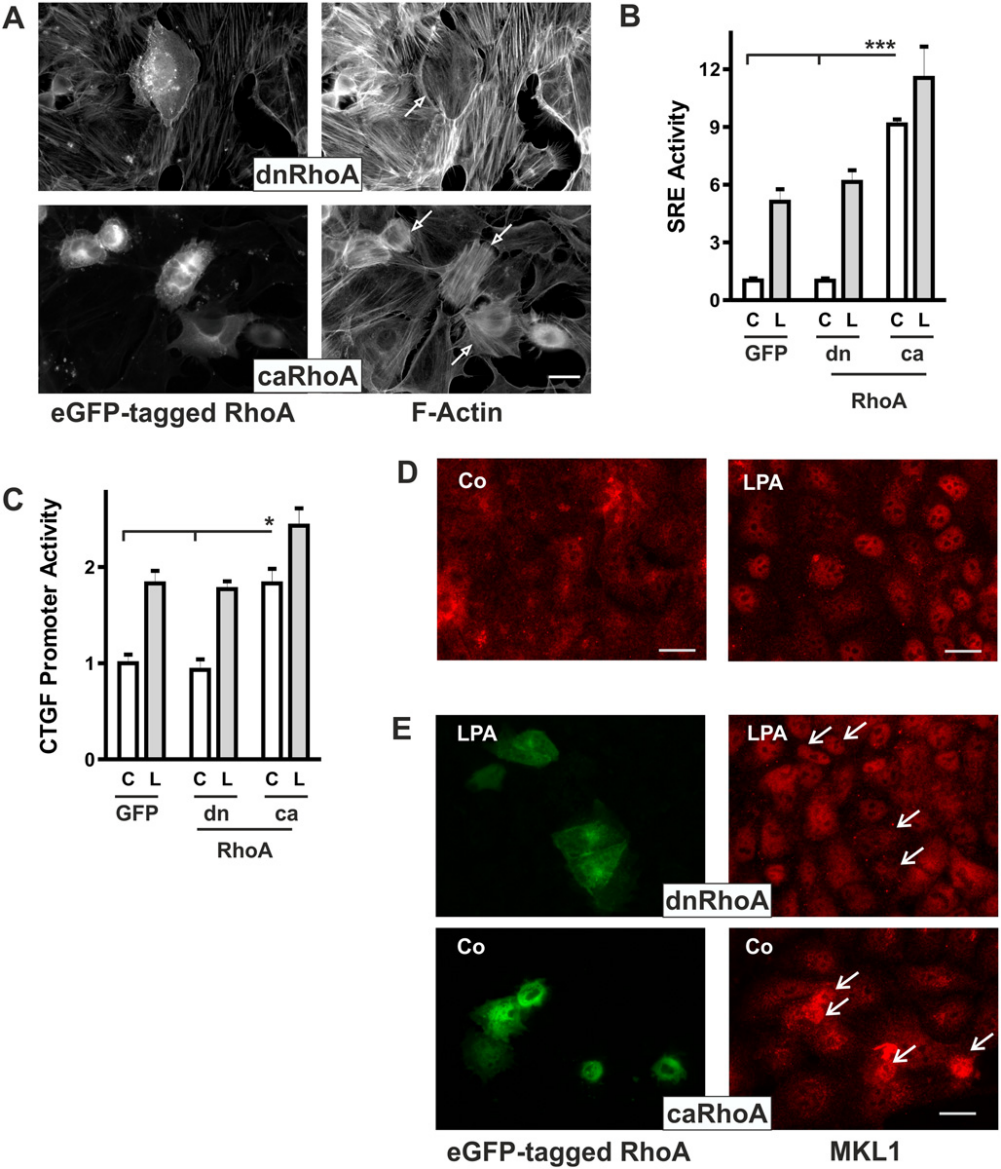
Overexpression of activated RhoA activates SRE activity and enhances CTGF promoter activity. (A) HKC-8 cells were transfected with constitutively active RhoA (G14V) or dominant negative RhoA (T19N) coupled to eGFP. 24 h after transfection, cells were stimulated with LPA (10 μM) for 1 h. Actin fibers were visualized with PromoFluor phalloidin. Arrows indicate transfected cells. Scale bar: 20 μm. (B) HKC-8 cells were transfected with eGFP-coupled dominant negative (dn) or constitutively active (ca) Rho GTPases, or eGFP (-) as control together with luciferase-coupled SRE constructs. Expression of cotransfected beta galactosidase was used as reference. Cells were stimulated with LPA (L, 10 μM) for 4 h. Data shown are means ± SD of 3 independent experiments performed with duplicate transfections. In each experiment the mean of the unstimulated control values was set to 1. SRE activity in caRhoA in control cells (C) was significantly increased (p < 0.001); ANOVA with Tukey’s multiple comparison test. (C) HKC-8 cells were transfected with eGFP-coupled constitutively active RhoA (caRhoA) or eGFP (Co) and luciferase-coupled 4.5 kb CTGF promoter constructs. Cotransfected Beta galactosidase was used as reference. Data are means ± SD of 3 experiments performed in duplicates. The mean values of control cells were set to 1. CTGF promoter activity was significantly higher in RhoA transfected cells (p < 0.05, ANOVA with Tukey’s multiple comparison test.) (D) HKC-8 cells were transfected with eGFP. 24 h after transfection the cells were stimulated with LPA for 1 h. MKL1 was detected by indirect immunofluorescence. Scale bar: 20 μm. (E) HKC-8 cells were transfected with eGFP-tagged dominant negative RhoA (T19N) constitutively active RhoA (G14V) (green) and stimulated with LPA for 1 h. Expression of MKL1 was detected by immunocytochemistry (red). Scale bar: 20 μm.

F-actin structures are strongly regulated by the interplay of Rho GTPases. Therefore, we analyzed the impact of overexpression of dominant negative and constitutively active human Rho GTPases on cell structure and gene expression in HKC-8 cells. Cells transfected with dnRhoA (pEGFP/RhoA(T19N)) lost cell spanning F-actin fibers (Fig. 2A). By contrast, caRhoA (pEGFP/RhoA(G14V)) induced a dense network of fine F-actin fibers, the cells rounded and tended to detach from the monolayer. While overexpression of both constructs markedly altered the cytoskeleton, only overexpression of constitutively active RhoA significantly increased SRE activity (Fig. 2B). Activation of SRE by caRhoA was comparable to the increase obtained by LPA stimulation. Overexpression of caRhoA also stimulated basal and LPA-stimulated CTGF promoter activity (Fig. 2C). While caRhoA induced an almost 10fold increase in SRE activity, the increase in CTGF promoter activity was moderate (about 2fold). The moderate relative increase was attributed at least in part to the higher baseline activity of the promoter which contains multiple binding sites for transcription factors active in cultured cells. As a link between LPA, RhoA and gene expression, the transcription factor MKL1 was analyzed. In control cells, MKL was distributed in nuclei and cytosol, whereas upon stimulation with LPA, nuclear localization prevailed (Fig. 2D). Nuclear localization was also observed in LPA-stimulated cells which were transfected with dnRhoA (Fig. 2E). However overexpression of caRhoA induced nuclear localization of MKL1 in control cells, confirming RhoA as an activator of MLK1. As expected [23], down-regulation of MKL1 by siRNA reduced LPA-mediated activation of SRE and induction of CTGF (S3 Fig.).

**Fig 3:**
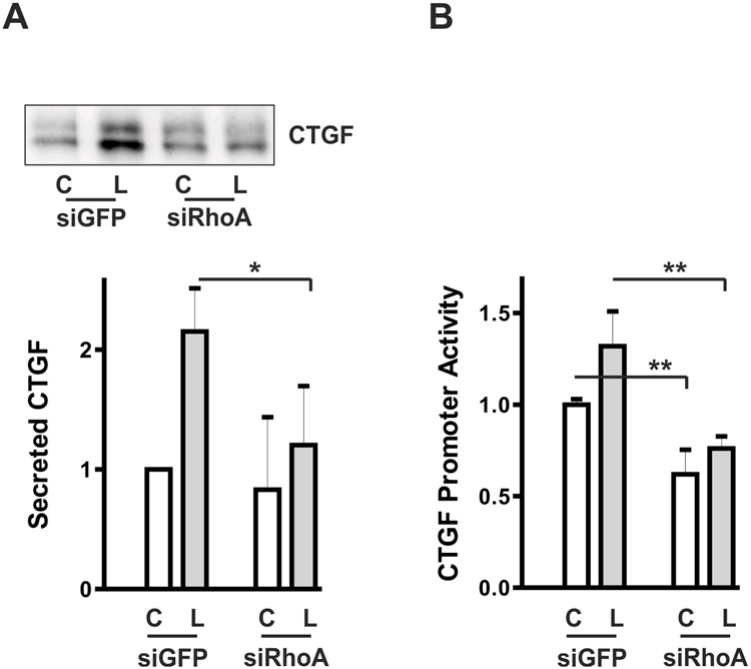
Transient down-regulation of RhoA interferes with CTGF synthesis. (A) HKC-8 cells were transfected with siRNA against GFP or RhoA. After 48 h, cells were stimulated with LPA (L, 10 μM) for 2 h. Secreted CTGF was precipitated from the cell culture supernatants and analyzed by Western blotting. The graph summarizes data of 4 independent experiments. CTGF expression in controls cells was set to 1 in each experiment. Means ± SD, * p < 0.05, ANOVA with Tukey’s multiple comparison test. (B) HKC-8 cells were treated with siRNA directed against GFP or RhoA 3 h after seeding. One day after siRNA transfection, HKC-8 cells were transfected with the 4.5 kb CTGF promoter construct. Stimulation with LPA was 4 h. The graph summarizes means ± SD of 3 independent experiments. Promoter activity in LPA-stimulated siGFP-transfected cells was set to 1 in each experiment. * *p < 0.001, ANOVA with Tukey’s multiple comparison test.

The missing inhibitory effect of dnRhoA on SRE activation and CTGF expression was unexpected. As a complementary approach to modulate RhoA activity, the GTPase was transiently down-regulated by siRNA. Under these conditions, LPA-induced CTGF secretion was significantly reduced (Fig. 3A). Interestingly basal CTGF secretion was also modulated by siRhoA. This became even more evident, when the promoter activity of CTGF was analyzed (Fig. 3B). A significant reduction of CTGF promoter activity not only in stimulated cells but also in cells cultured without specific stimulation showed that RhoA played an essential role in CTGF regulation. Thus, even though overexpression of dnRhoA clearly altered cell morphology it was not sufficiently effective to reduce CTGF gene expression, while down-regulation of RhoA by siRNA demonstrated regulation of CTGF via this pathway.

### Differential effects of pharmacological inhibition and down-regulation of Rho kinases on LPA-induced activation of CTGF

**Fig 4:**
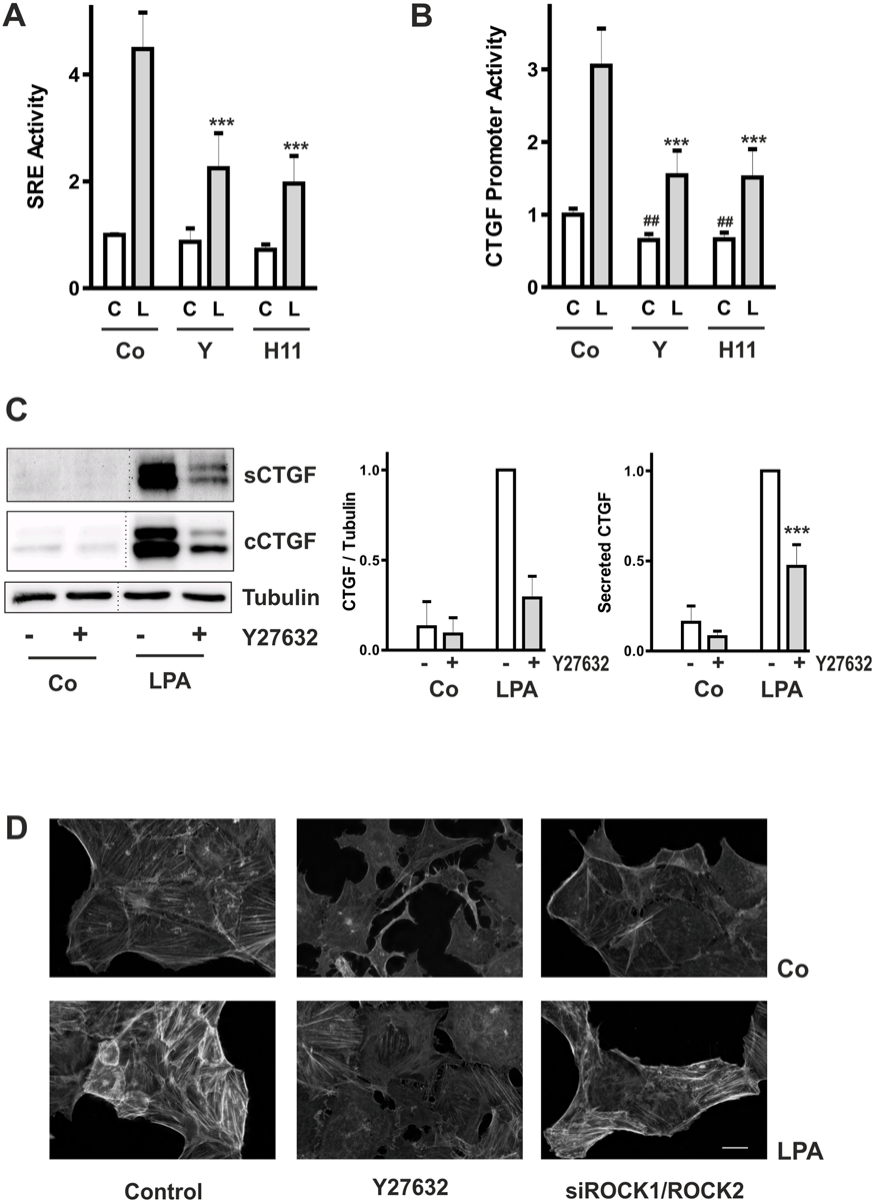
CTGF expression is dependent on Rho kinase activity. (A) HKC-8 cells were transfected with luciferase-coupled SRE constructs. Expression of transfected beta galactosidase was used as reference. Cells were preincubated for 30 min with Y27632 (Y, 10 μM) or H1152 (0.75 μM) and were then stimulated with LPA (L, 10 μM, grey bars) for 4 h. Data are means ± SD of 3 experiments with duplicate samples. In each experiment, means of control values were set to 1. *** p < 0.001 compared to LPA-stimulated control cells; ANOVA with Tukey’s multiple comparison test. (B) HKC-8 cells were treated as in A, but transfected with a 4.5 kb CTGF promoter construct. ## p < 0.01 compared to non-stimulated control cells, *** p < 0.001, compared to LPA-stimulated cells, ANOVA with Tukey’s multiple comparison test. (C) HKC-8 cells were preincubated with Y27632 (10 μM) for 30 min and then incubated with LPA (10 μM) for 2 h. CTGF was detected by Western blotting in the cell culture supernatants (secreted CTGF) and homogenates (cellular CTGF). Samples were detected on one blot which had to be rearranged (dotted line). The graphs summarize quantification of multiple experiments with LPA-stimulated cells set to 1 in each experiment: cellular CTGF: n = 2 ± half range; secreted CTGF control: n = 3, secreted CTGF LPA-stimulated: n = 6; *** p<0.001 compared to LPA-stimulated cells, ANOVA with Tukey’s multiple comparison test. (D) HKC-8 cells were transfected with siGFP or siROCK1/ROCK2 at day 1, incubated with Y27632 (10 μM) at day 2 and stimulated with LPA (10 μM) for 1 h at day 3. F-actin was visualized with PromoFluor phalloidin. Scale bar: 20 μm.

Stabilization of F-actin fibers downstream of RhoA is controlled by Rho kinases. Two chemically distinct inhibitors of Rho kinases, Y27632 and H1152, reduced the LPA-stimulated SRE activity by about 50% (Fig. 4A), although to a lesser extent than overexpression of mutated actin (R62D) (Fig. 1B). Similarly, both inhibitors reduced CTGF promoter activity (Fig. 4B). Reduced activity was also observed in the absence of LPA stimulation indicative of a contribution of Rho kinases to the basal activity of the CTGF promoter in cultured cells. Inhibition of CTGF transcription resulted in reduced synthesis of CTGF which was detected in cell homogenates and cell culture supernatants by Western blot analysis (Fig. 4C). Moreover, cells treated with Y27632 were void of cell spanning F-actin fibers, and only few fibers were seen upon stimulation with LPA (Fig. 4D). In a previous study we showed that down-regulation of Rho kinase isoforms ROCK1 and ROCK2 by siRNAs resulted in distinct morphological changes in HKC-8 cells [12]. Silencing of both isoforms led to morphological alterations comparable to those obtained by pharmacological inhibition of Rho kinases. By contrast, F-actin fiber formation by LPA was only partially prevented by siRNA treatment.

**Fig 5:**
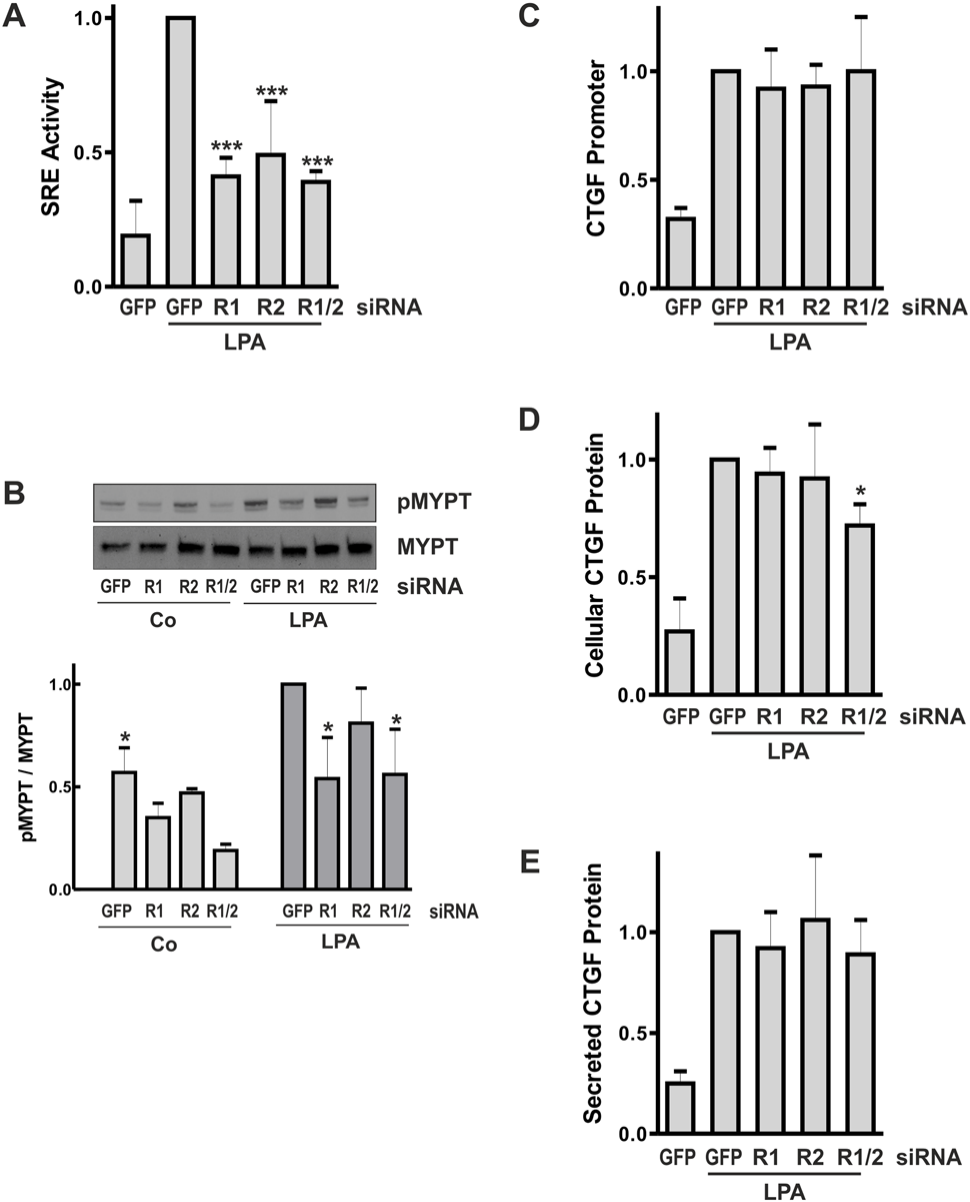
Transient down-regulation of Rho kinases is not sufficient to impair CTGF expression. HKC-8 cells were treated with siRNA directed against GFP, ROCK1 (R1) or ROCK2 (R2), or a combination of both (R1/2) 3 h after seeding. (A) One day after siRNA transfection, HKC-8 cells were transfected with SRE constructs. Relative luciferase activity was determined in control cells and cells stimulated with LPA for 4 h. The graph summarizes means ± SD of 3 to 5 independent experiments. SRE activity in cells stimulated with LPA was set to 1 in each experiment. Statistics were calculated for LPA-stimulated samples. *** p< 0.001, ANOVA with Tukey’s multiple comparison. (B) Phosphorylated MYPT (pMYPT) was detected by Western blot analysis in control cells and in cells stimulated with LPA for 3–5 min 48 h after siRNA treatment. Detection of total MYPT was used as control. The graph summarizes data of 3 experiments (mean ± SD). Expression of pMYPT/MYPT in LPA-stimulated siGFP-transfected cells was set to 1 in each experiment; * p<0.05 compared to LPA-stimulated siGFP-transfected cells. (C) One day after siRNA transfection, HKC-8 cells were transfected with CTGF promoter constructs. Stimulation with LPA was 4 h. The graph summarizes means ± SD of 3–4 independent experiments. Promoter activity in LPA-stimulated siGFP-transfected cells was set to 1 in each experiment. (D) CTGF protein was detected in cellular homogenates. Data are means ± SD of 3–5 independent experiments. CTGF protein detected in LPA-stimulated cells was set to one in each experiment. * p<0.05 compared to LPA-stimulated siGFP-transfected cells. (E) CTGF protein was detected in cell culture supernatants. Data are means ± SD of 3–4 independent experiments.

Down-regulation of either ROCK1 or ROCK2 significantly reduced LPA-stimulated SRE activity by more than 50% (Fig. 5A). However, in terms of endogenous biological activity, differences between ROCK1 and ROCK2 became evident. Down-regulation of ROCK1 significantly reduced basal and LPA-stimulated phosphorylation of MYPT, one of the downstream targets of Rho kinases, whereas down-regulation of ROCK2 was less effective (Fig. 5B).

Based on the strong inhibition of SRE activity by inhibition of Rho kinase activity, reduction of CTGF promoter activity and CTGF protein synthesis was expected. However, neither the CTGF promoter activity nor the amount of secreted protein was significantly reduced by down-regulation of either or both isoforms of Rho kinase (Fig. 5C/E). Simultaneous inhibition of both isoforms resulted in a moderate inhibition of intracellular CTGF protein (Fig. 5D), an effect which was observed in some but not all promoter analyses (Fig. 5C).

Whereas comparable effects of pharmacological inhibition of Rho kinases and siRNA-mediated down-regulation were observed at the level of SRE promoter activity, profound differences were observed in terms of endogenous CTGF regulation.

### Rac1 signaling in proximal tubular epithelial cells

**Fig 6:**
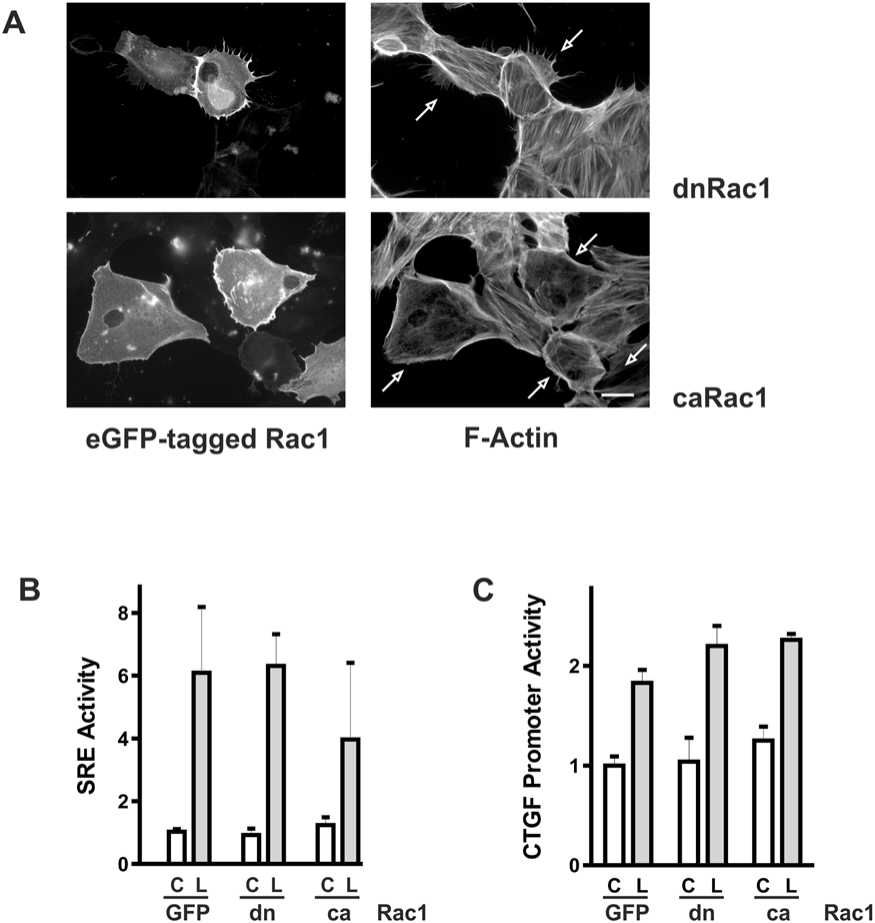
Overexpression of activated or dominant negative Rac1 alters cell morphology but not CTGF expression. (A) HKC-8 cells were transfected with dominant negative Rac1 (T17N) or constitutively active Rac1 (G12V) coupled to eGFP. 24 h after transfection, cells were stimulated with LPA (10 μM) for 1 h. F-actin fibers were visualized with PromoFluor phalloidin. Arrows indicate transfected cells. Scale bar: 20 μm. (B) HKC-8 cells were transfected with eGFP-coupled dominant negative (dn) or constitutively active (ca) Rac1, or eGFP (GFP) as control together with luciferase-coupled SRE constructs. Expression of cotransfected beta galactosidase was used as reference. Cells were stimulated with LPA (L, 10 μM) for 4 h. Data shown are means ± SD of 3–5 independent experiments performed with duplicate transfections. In each experiment the mean of the unstimulated control values was set to 1. (C) Cells were treated as in (B) but transfected with a 4.5 kb CTGF promoter construct. Data are means of 3 independent experiments with duplicate transfections. In each experiment the mean of the unstimulated control values was set to 1.

Besides RhoA-induced signaling, Rac1 is an essential mediator of alterations of the actin cytoskeleton. Cells transfected with dnRac1 (pEGFP/Rac1(T17N)) presented extended spikes, whereas cells transfected with caRac1 (pEGFP/Rac1(G12V)) flattened and were essentially void of cell spanning F-actin fibers (Fig. 6A). Even though alterations of the F-actin cytoskeleton were evident overexpression of neither Rac1 mutant significantly altered SRE activity (Fig. 6B) or CTGF promoter activity (Fig. 6C).

**Fig 7:**
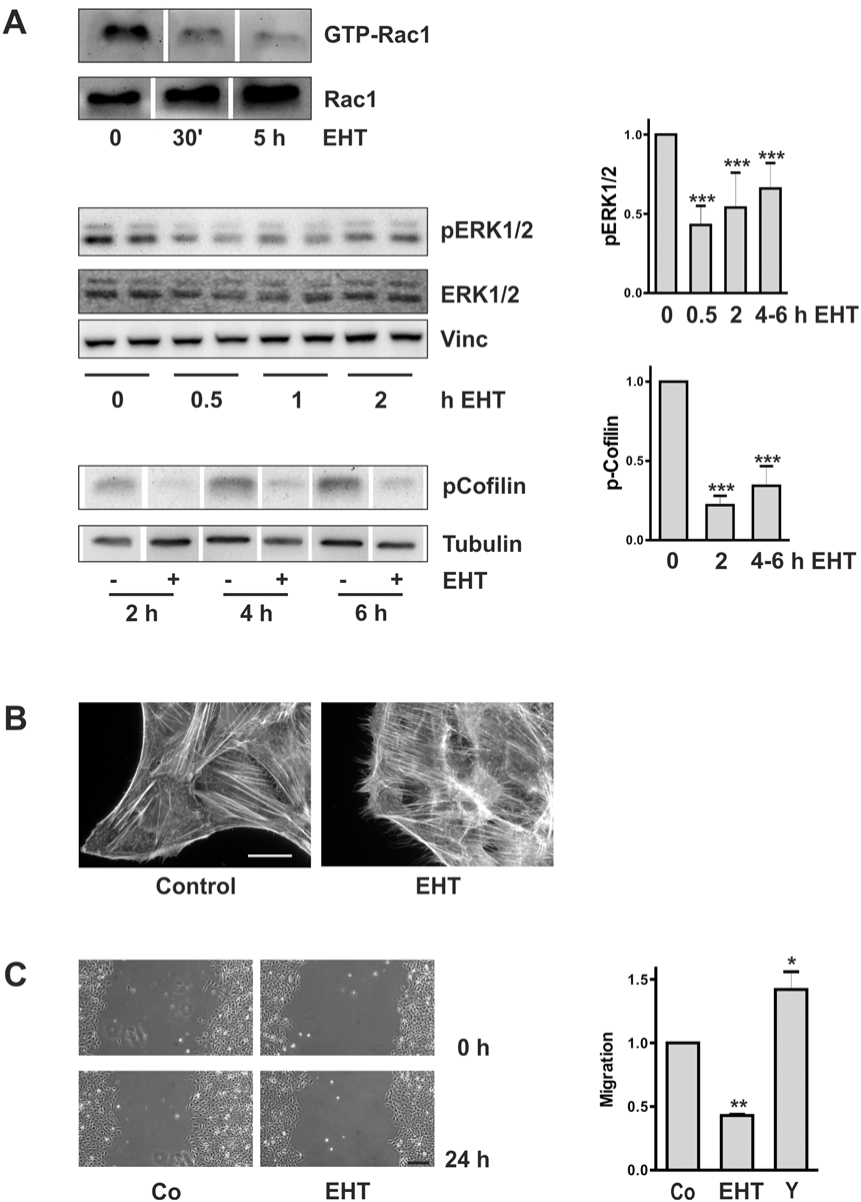
Cellular effects of Rac1 inhibition by EHT1864. (A) HKC-8 cells were incubated with EHT1864 (10 μM) for the times indicated. Rac1 activity was determined by pull down experiments. The blot is representative of 3 experiments with comparable results. Samples were run on one blot which had to be rearranged. pERK1/2, ERK1/2 and vinculin were detected by immunoblot procedure. The blot shows duplicate biological samples. The graph summarizes data of n = 3–6 experiments (pERK1/2 / ERK1/2 or pERK1/2 / vinculin), means ± SD, *** p<0.001, compared to control cells, ANOVA with Dunnett’s multiple comparison test. pCofilin and tubulin were detected on one blot which had to be rearranged. The graph summarizes data of n = 4 experiments (pCofilin/Tubulin), means ± SD, *** p<0.001, compared to control cells, ANOVA with Dunnett’s multiple comparison test. (B) HKC-8 cells were treated with EHT1864 (10 μM) for 30 min. F-actin was visualized with PromoFluor phalloidin. Scale bar: 20 μm. (C) HKC-8 cells were seeded around barriers. After removal of the barriers (t = 0 h) the cells were treated with EHT1864 (10 μM) for 24 h. Scale bar: 200 μm. The graph summarizes the relative migration velocity of cells treated with 5 or 10 μM EHT1864 or 10 μM Y27632. Data are means ± SEM of 3 experiments with 4 determinations each. ** p< 0.01, * p<0.05 compared to control cells.

To further address Rac1 signaling in HKC-8 cells the activity of Rac1 was inhibited by the specific low molecular weight inhibitor EHT1864, which blocks the GTP binding site [24]. Treatment of the cells with 10 μM EHT1864 reduced Rac1 activity as determined by pull down assays (Fig. 7A). Furthermore, it reduced phosphorylation of known effector proteins of Rac1, namely phosphorylation of ERK1/2 and Cofilin (Fig. 7A). The morphology of the cells was reminiscent of the appearance of cells transfected with dnRac1, characterized by F-actin spikes (Fig. 7B). Functionally, EHT1864 prevented cell migration (Fig. 7C) which was stimulated in control cells in the presence of the Rho kinase inhibitor Y27632 in line with published results [25].

### Complex regulation of CTGF expression by Rac1 inhibition

**Fig 8:**
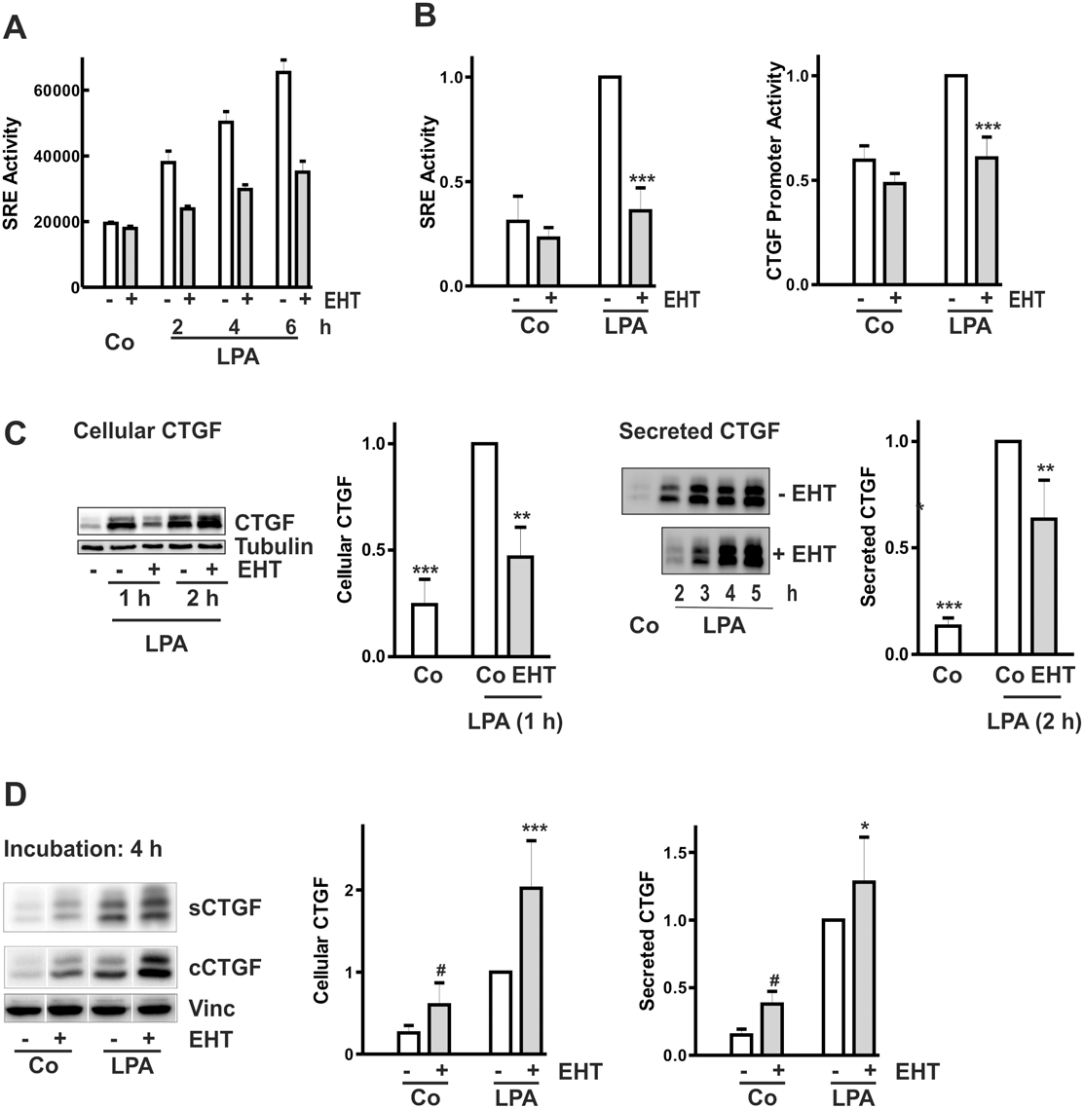
Time-dependent effects of Rac1 inhibition by EHT1864 on CTGF regulation. (A) HKC-8 cells were transfected with SRE promoter constructs. After pre-incubation with EHT1864 (10 μM) for 30 min, the cells were stimulated with LPA (10 μM) for the times indicated. Data are means ± SD from one experiment with triplicate transfections. (B) HKC-8 cells were transfected with SRE or 4.5 kb CTGF promoter constructs. Relative luciferase activity was determined in control cells and cells pre-treated with EHT1864 (10 μM) for 30 min and then stimulated with LPA (10 μM) for 3–4 h. Data are means ± SD of 7 (SRE) and 3 (CTGF promoter) experiments. Activity in LPA-stimulated cells was set to 1 in each experiment. *** p<0.001, compared to cells stimulated with LPA, ANOVA with Dunnett’s multiple comparison test. (C) HKC-8 cells were pre-incubated with EHT1864 (10 μM) for 30 min and then stimulated with LPA (10 μM) for the times indicated. Cellular CTGF was determined in preparations of cellular homogenates by Western blotting. Tubulin was used to control for equal blotting and detection. The graph summarizes data of n = 3 experiments, stimulated with LPA for 1 h (CTGF/tubulin, means ± SD). Secreted CTGF was determined in precipitates of cell culture supernatants by Western blotting. The graph summarizes data of n = 4 experiments, stimulated with LPA for 2 h. Data were normalized to LPA-stimulated CTGF synthesis. *** p<0.001, ** p<0.01, ANOVA with Tukey’s multiple comparison test. (D) HKC-8 cells were pre-incubated with EHT1864 (10 μM) for 30 min and then stimulated with LPA (10 μM) for 4 h. Secreted CTGF (sCTGF) was determined in precipitates of cell culture supernatants and cellular CTGF (cCTGF) in cellular homogenates by Western blotting. Vinculin (vinc) served as control. Samples were detected on one blot which had to be rearranged. Data of 4–5 experiments (cellular CTGF, 4 h) and 5–7 experiments (secreted CTGF, 4–6 h) are summarized in the graphs. CTGF expression in LPA-stimulated cells was set to 1 in each experiment. * p<0.05, ***p<0.001 compared to LPA-stimulated cells, ANOVA with Tukey’s multiple comparison test. # p<0.05, paired 2-sided t-test, EHT1864-treated cells compared to control cells.

Inhibition of Rac1 by EHT1864 interfered with the LPA-induced increase of SRE activity and also reduced CTGF promoter activity (Fig. 8A/B). Promoter activities were reduced by the inhibitor during the time course of the measurement for up to 6 h. However, when LPA-induced cellular CTGF synthesis was analyzed, a significant inhibition of CTGF synthesis was observed only at the 1 h time point (Fig. 8C). This was also reflected at the level of secreted CTGF, where a significant inhibition was detectable after 2 h (Fig. 8C). At 4 h of incubation with EHT1864, however, increased CTGF synthesis became obvious in control cells. The increase was detectable in cellular homogenates reflecting ongoing synthesis of CTGF (Fig. 8D). Furthermore, LPA-mediated CTGF synthesis was no longer inhibited but rather increased most prominently detected in cellular homogenates (Fig. 8D).

**Fig 9:**
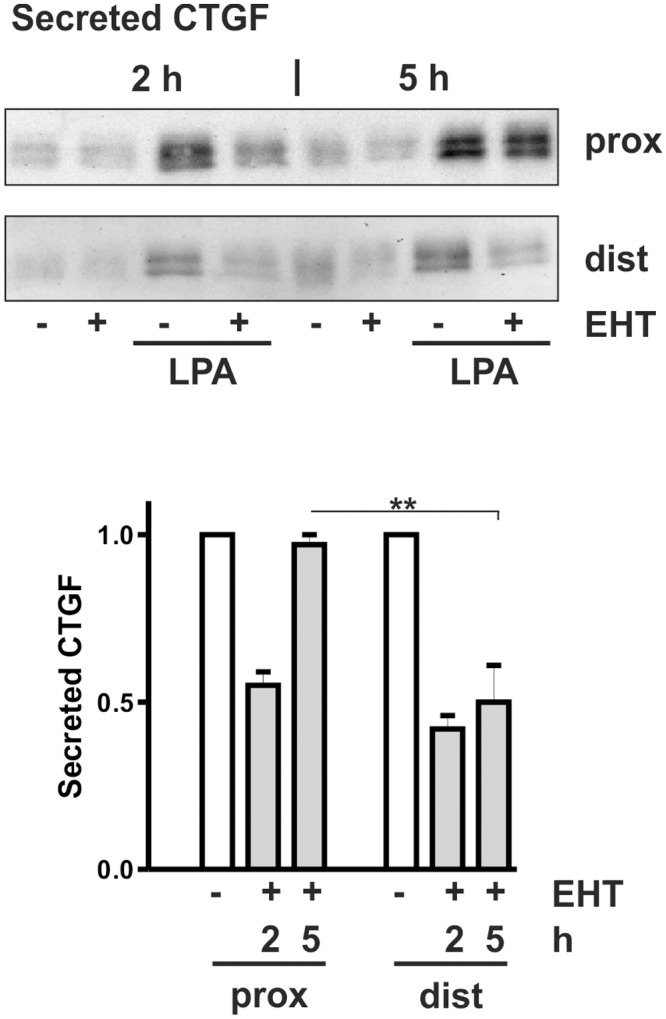
Differential regulation of CTGF by EHT1864 in epithelial cells of proximal and distal origin. Primary proximal and distal tubular epithelial cells were pre-incubated with EHT1864 (10 μM) for 30 min and then incubated with LPA (10 μM) for 2 and 5 h. Secreted CTGF was detected in the cell culture supernatants. Data are means of 3 (2 h) and 4 (5 h) preparations of cells obtained from different patients. Secretion of control cells was set to one in each experiment. ** p < 0.01, two-sided Student’s t-test.

The dual regulation of CTGF by EHT1864 was confirmed in isolates of human primary tubular epithelial cells. Human tubular epithelial cells of proximal and distal origin can be distinguished by their expression of the cell-cell adhesion molecules, N-cadherin and E-cadherin, respectively [11]. Based on these criteria, proximal cell preparations contained over 60% proximal cells whereas distal cells were > 90% E-cadherin positive. Short term incubation with EHT1864 inhibited CTGF secretion in both cell populations. Prolonged incubation, however, reduced CTGF only in preparations with distal cells (Fig. 9). In proximal cells, incubation with EHT1864 led to the same regulation as observed in HKC-8 cells with inhibition being only detectable in the early stimulation phase. The dual regulation of CTGF by inhibition of Rac1 activity by EHT1864 thus seems to be restricted to proximal tubular epithelial cells.

## Discussion

Manipulation of G-actin levels by enforced expression of actin mutants profoundly altered the cell morphology of the renal proximal tubular cells investigated in this study, and also modulated MKL1—SRF signaling. Activation of the CArG box/SRE element was regulated by both, RhoA and Rac1, as shown by pharmacological inhibition of either RhoA-Rho kinase or Rac1 signaling. The importance of these signaling pathways was also reflected at the level of CTGF promoter activity and CTGF synthesis. However, the more detailed analysis performed in this study revealed the complexity of the regulation of CTGF gene expression related to alterations of the actin cytoskeleton: overexpression of dominant negative RhoA or Rac1 proteins allowed the cells to compensate for the inhibition of the GTPases, whereas RhoA siRNA or pharmacological inhibition of Rho kinases markedly reduced CTGF synthesis. Pharmacological inhibition of Rac1 only transiently inhibited CTGF synthesis followed by an increased CTGF synthesis. Down-regulation of individual Rho kinase isoforms was sufficient to reduce SRE activation but failed to interfere with CTGF promoter activation or CTGF synthesis. These results correspond to a time dependent dynamic regulation of gene expression provoked and/or accompanied by morphological alterations of the F-actin cytoskeleton.

Our data also point to cell type specific regulation. While it is obvious to expect differences between mesenchymal and epithelial cells, our data imply differences among various types of epithelial cells. Proximal tubular epithelial cells differ strongly from distal tubular cells related to cell-cell and cell-matrix adherence and in their ability to undergo mesenchymal alterations [12]. Our results obtained with the proximal cell line HKC-8 cells and primary proximal cells differ from data obtained with the MDCK cell line which was derived from canine distal tubular cells: Rac1 as opposed to RhoA was defined in those cells as major regulator of SRE activity modulated by E-cadherin cell-cell contacts [9, 26]. Instead of E-cadherin, HKC-8 cells express N-cadherin as major cell-cell adhesion molecule. There is ample evidence for cadherins being differentially involved in cellular signaling [27]. However, the role of N-cadherin has not yet been addressed directly in proximal tubular cells and it cannot be excluded that additional factors contribute to the difference between proximal and distal epithelial cells. Comparison of renal cells with epithelial cells obtained from other organs might shed light on epithelial cell type specific regulation of gene expression by changes in the cytoskeletal architecture.

Based on the results of overexpression of dominant negative and constitutively active RhoA and Rac1 constructs, there seemed to be a clear dominance of RhoA signaling in terms of SRE activity and CTGF expression. Overexpression of caRhoA not only increased F-actin fibers but also reduced attachment of the cells, implicating alterations of cell-cell contacts. This may also modulate Src activity [28], which has been shown earlier to regulate CTGF in HKC-8 cells [29]. Corresponding data were obtained by down-regulation of RhoA by siRNA, which strongly reduced basal and LPA-stimulated CTGF synthesis supporting the notion of an actin-dependent regulation of CTGF.

As downstream mediators of RhoA signaling, Rho kinases were investigated which are critically involved in CTGF regulation in various cell types [6]. While there is clear genetic and functional evidence for specific roles of Rho kinase isoforms, these differences are not observed in all cellular systems [30]. In earlier studies we observed differential changes of F-actin structures and cell morphology by siROCK1 and siROCK2 in HKC-8 cells and primary proximal tubular epithelial cells [12]. Isoform-specific effects were observed in this study at the level of MYPT phosphorylation, whereas down-regulation of either isoform was functionally effective in reduction of SRE activity. However, ROCK down-regulation was not reflected comparably at the level of CTGF synthesis. Obviously other signaling pathways were sufficiently activated in siRNA-treated cells to compensate for the reduced Rho kinase signaling. This was also evident at the morphological level, where down-regulation of Rho kinases only partially prevented LPA-induced formation of F-actin stress fibers, which was abrogated in Y27632-treated cells. The formin mDIA2 has recently been shown to affect MKL-dependent nuclear activity [31]. This may also play a role in epithelial cells because high concentrations of Rho kinase inhibitors (Y27632, 10 μM and H1152, 0.75 μM) only partially inhibited CTGF in HKC-8 cells, whereas complete inhibition was seen in mesenchymal cells such as endothelial cells [32].

Based on the overexpression of dnRac1 or caRac1, Rac1 signaling did not seem to be relevant for CTGF expression in HKC-8 cells. Pharmacological inhibition of Rac1, however, revealed a more complex regulation. In the early phase, there was a prominent inhibition of CTGF, whereas at later time points, a stimulation of CTGF synthesis prevailed. Consistent with this dynamic regulation, there was no significant effect in HKC-8 cells which had been transfected with dnRac1 for 24 to 48 h suggesting adaptation of the cells to Rac1 reduction. Dynamic regulation was also observed in primary proximal cells whereas a prolonged inhibition of CTGF expression was detected in human distal tubular epithelial cells, reminiscent of the role for Rac1 in SRF activation described in MDCK cells [9]. Thus far, Rac1 inhibition has been related to CTGF expression primarily in mesenchymal cells such as fibroblasts [10, 33], cardiomyocytes [34] or renal mesangial cells [35]. In all these studies, pharmacological or genetic inhibition of Rac1 correlated with reduced CTGF expression. However, the molecular mechanism of the unique dual regulation of CTGF expression after pharmacological inhibition of Rac1 activity described here for proximal tubular epithelial cells needs further investigation. It may well depend on signaling pathways unrelated to SRE signaling. We have shown earlier that mitogen-activated kinases play a role in the regulation of CTGF in renal tubular epithelial cells [36], which were not addressed in this manuscript.

Our studies show profound differences in the functional outcome of interference with GTPase activities by pharmacological or by genetic manipulation of Rho GTPase signaling. Neither overexpression of dnRac1 nor caRac1 affected SRE or CTGF promoter activity whereas both were strongly reduced by the Rac1 inhibitor. Similarly, pharmacological inhibition of Rho kinases strongly inhibited promoter activities and CTGF synthesis whereas down-regulation of Rho kinases barely affected regulation of CTGF expression. Different factors may contribute to these differences: Genetic manipulation of the cells implies long term alterations not only of Rho GTPase activity but also of protein content. Thus, not only is GTPase signaling affected but also are protein-protein interactions perturbed due to lack of proteins. This aspect may contribute to the discrepancy between treatment of the cells with dnRhoA and siRhoA. Similar discrepancies between overexpression and siRNA approaches have also been noted in other systems [37] and seem to give raise to a more general caveat to the methods of cell manipulation, especially related to proteins which are major regulators. As an additional aspect, long term interference, 24 to 48 h, may allow the cells to adapt to alterations in protein synthesis. Feedback loops have been described between Rho and Rac [27], also involving downstream mediators, e.g. modulation of Rac1 and RhoA by inhibition of ROCK1 [38].

Regulation of the CArG box element by alterations of the cytoskeleton largely reflected modulation of CTGF expression in tubular epithelial cells. However, translation of morphological alterations into gene expression was not restricted to CArG box/SRE activation, but was modulated by additional regulatory pathways. These become particularly noticeable when cells are allowed to adapt to interference with particular signaling pathways. This reasoning may explain discrepancies often observed between short term in vitro experiments and genetic manipulation in vivo.

## Supporting Information

S1 Fig Efficiency of siRNA knockdown. HKC-8 cells were treated with 20 nM siRNA as described in the methods section. Western blots were performed after 48 h. The size of the bands detected was controlled by molecular weight standards run on the same gel. A: HKC-8 cells were treated with siRNA directed against ROCK1 and ROCK2. Separate blots were performed to detect ROCK1 and ROCK2. Tubulin was used to confirm equal loading and blotting. B: HKC-8 cells were treated with siRNA directed against RhoA. Tubulin was used to confirm equal loading and blotting. C: HKC-8 cells were treated with siRNA directed against MKL1. Expression of MKL1 in siRNA-treated cells was too low to allow quantification.
(TIF)

S2 Fig Morphological alterations caused by overexpression of actin mutants in HKC-8 cells. HKC-8 cells were transfected with mutated actin R62D or S14C for 24 h and then stimulated with LPA (10 μM) for 1 h. Flag-tagged actin was detected by indirect immunofluorescence and actin fibers were visualized by PromoFluor phalloidin. Scale bars: 20 μm.
(TIF)

S3 Fig MKL1 is involved in SRE and CTGF regulation in LPA-stimulated HKC-8 cells. (A) HKC-8 cells were treated with siRNA directed against MKL1 or scrambled siRNA and then transfected with an SRE construct the following day. After 24 h, cells were stimulated with LPA for 3 h and SRE luciferase activity was detected after 3 h. Data are means ± SD of triplicate transfections. (B) HKC-8 cells were treated with siRNA directed against MKL1 or GFP at day 1. After 48 h, cells were stimulated with LPA for 2 h. Secreted CTGF was detected in the cell culture supernatants by Western blotting.
(TIF)
