# Supplementary figures and images for: Actin-Mediated Gene Expression Depends on RhoA and Rac1 Signaling in Proximal Tubular Epithelial Cells
Source: PLoS One. 2015 Mar 27;10(3):e0121589. doi: 10.1371/journal.pone.0121589 (PMC4376694; doi:10.1371/journal.pone.0121589)

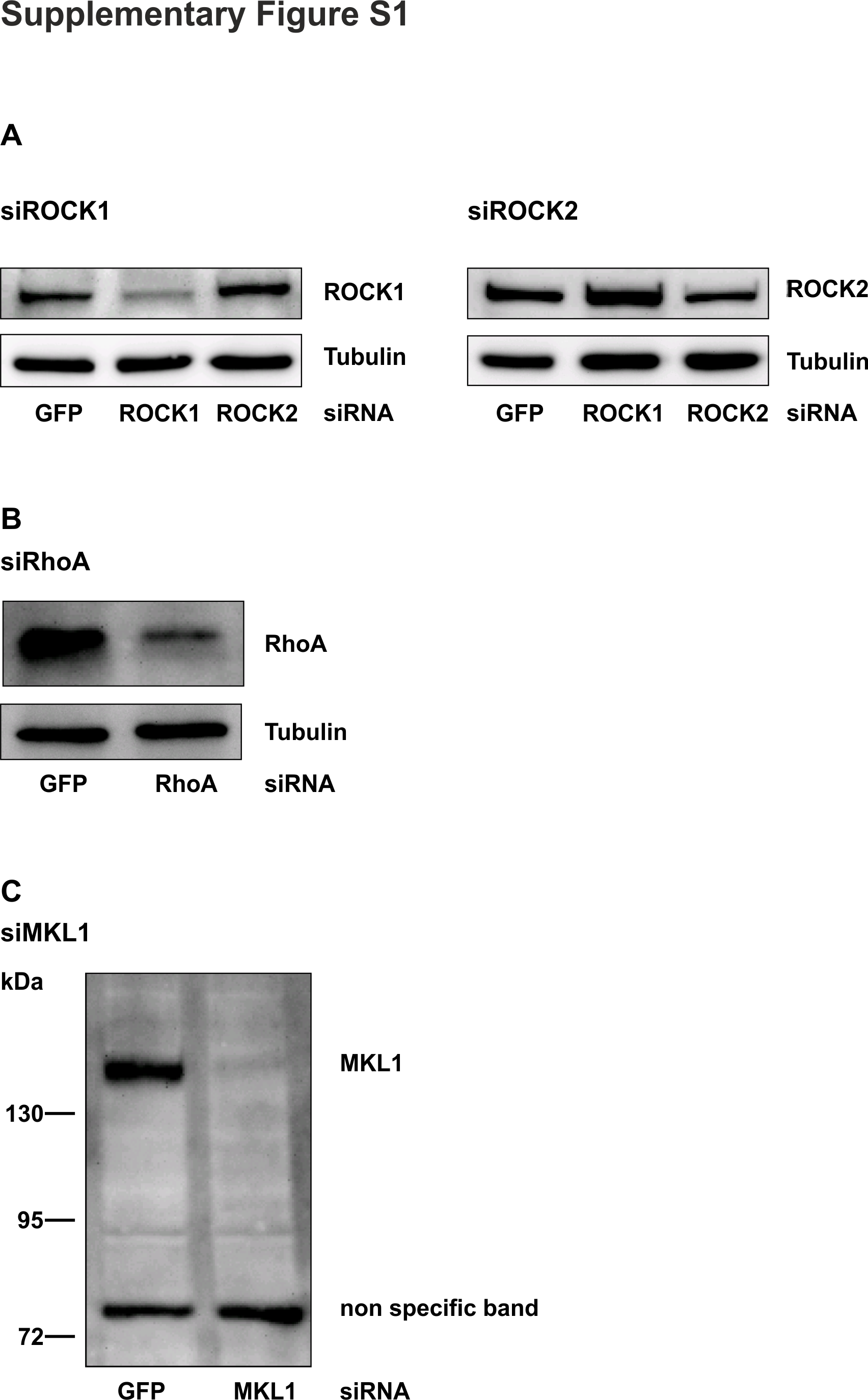

Supplement: S1 Fig — HKC-8 cells were treated with 20 nM siRNA as described in the methods section. Western blots were performed after 48 h. The size of the bands detected was controlled by molecular weight standards run on the same gel. A: HKC-8 cells were treated with siRNA directed against ROCK1 and ROCK2. Separate blots were performed to detect ROCK1 and ROCK2. Tubulin was used to confirm equal loading and blotting. B: HKC-8 cells were treated with siRNA directed against RhoA. Tubulin was used to confirm equal loading and blotting. C: HKC-8 cells were treated with siRNA directed against MKL1. Expression of MKL1 in siRNA-treated cells was too low to allow quantification. (TIF) [file pone.0121589.s001.tif]

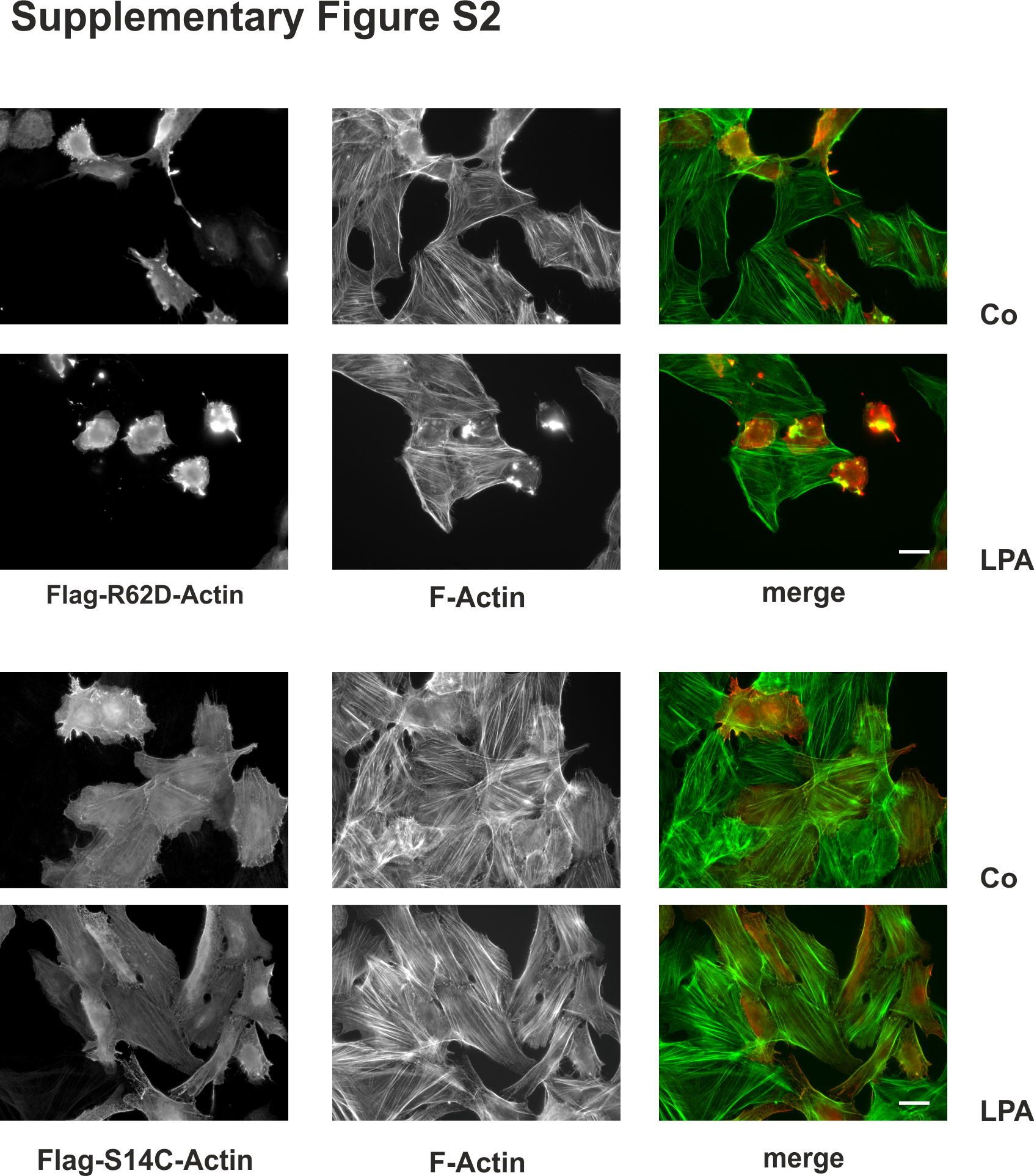

Supplement: S2 Fig — HKC-8 cells were transfected with mutated actin R62D or S14C for 24 h and then stimulated with LPA (10 μM) for 1 h. Flag-tagged actin was detected by indirect immunofluorescence and actin fibers were visualized by PromoFluor phalloidin. Scale bars: 20 μm. (TIF) [file pone.0121589.s002.tif]

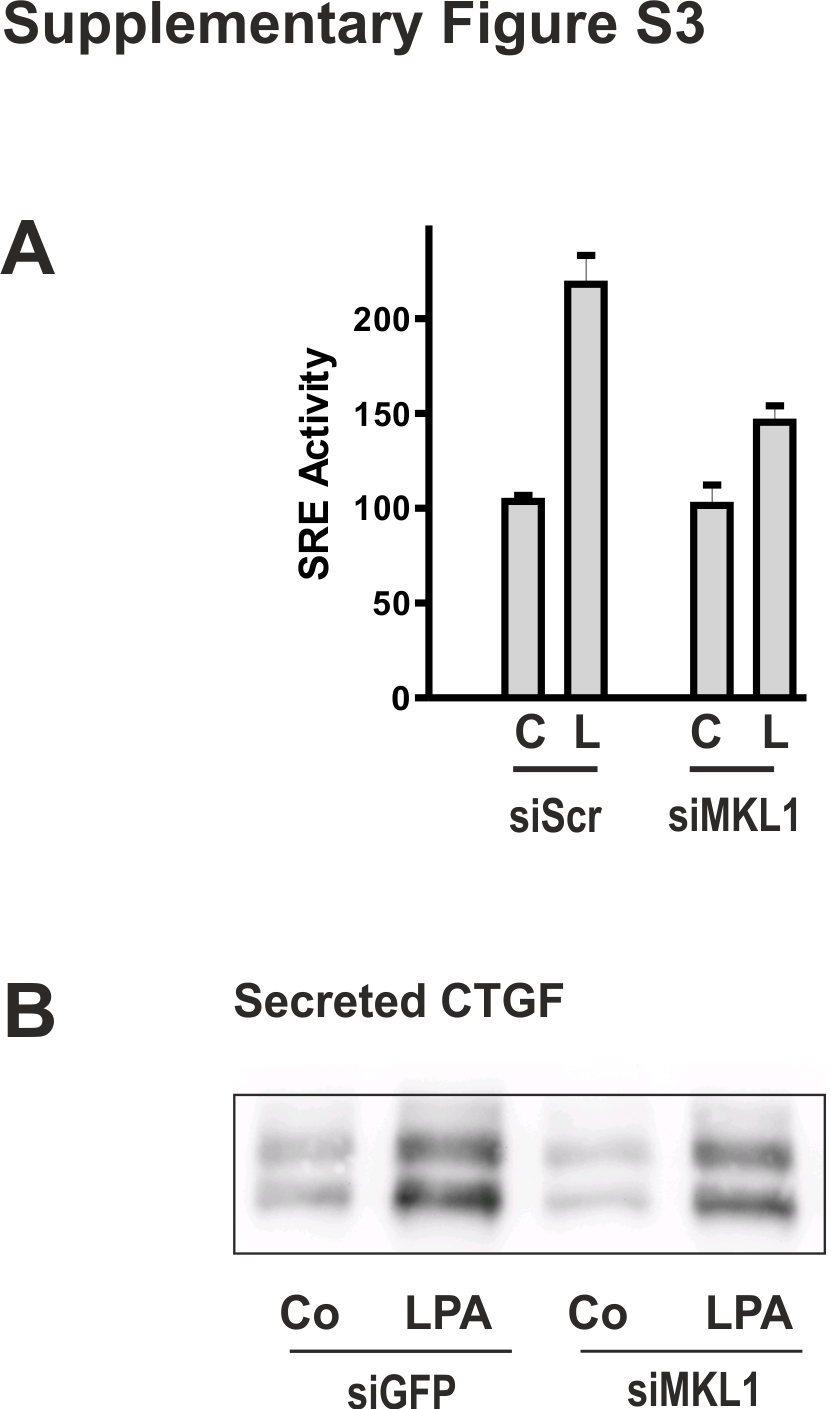

Supplement: S3 Fig — (A) HKC-8 cells were treated with siRNA directed against MKL1 or scrambled siRNA and then transfected with an SRE construct the following day. After 24 h, cells were stimulated with LPA for 3 h and SRE luciferase activity was detected after 3 h. Data are means ± SD of triplicate transfections. (B) HKC-8 cells were treated with siRNA directed against MKL1 or GFP at day 1. After 48 h, cells were stimulated with LPA for 2 h. Secreted CTGF was detected in the cell culture supernatants by Western blotting. (TIF) [file pone.0121589.s003.tif]
